# Soluble Angiotensin Converting Enzyme 2 (ACE2) Is Upregulated and Soluble Endothelial Nitric Oxide Synthase (eNOS) Is Downregulated in COVID-19-induced Acute Respiratory Distress Syndrome (ARDS)

**DOI:** 10.3390/ph14070695

**Published:** 2021-07-19

**Authors:** Alice G. Vassiliou, Alexandros Zacharis, Chrysi Keskinidou, Edison Jahaj, Maria Pratikaki, Parisis Gallos, Ioanna Dimopoulou, Anastasia Kotanidou, Stylianos E. Orfanos

**Affiliations:** 1 First Department of Critical Care Medicine & Pulmonary Services, School of Medicine, National and Kapodistrian University of Athens, Evangelismos Hospital, 106 76 Athens, Greece; alvass75@gmail.com (A.G.V.); alexandroszacharis5@gmail.com (A.Z.); chrysakes29@gmail.com (C.K.); edison.jahaj@gmail.com (E.J.); idimo@otenet.gr (I.D.); akotanid@med.uoa.gr (A.K.); 2 Biochemical Department, Evangelismos Hospital, 106 76 Athens, Greece; marypratik@icloud.com; 3 Computational Biomedicine Laboratory, Department of Digital Systems, University of Piraeus, 185 34 Piraeus, Greece; parisgallos@yahoo.com

**Keywords:** eNOS, sACE2, COVID-19, adrenomedullin, NO, treatment

## Abstract

A damaged endothelium is an underlying condition of the many complications of COVID-19 patients. The increased mortality risk associated with diseases that have underlying endothelial dysfunction, such as acute respiratory distress syndrome (ARDS), suggests that endothelial (e) nitric oxide synthase (NOS)-derived nitric oxide could be an important defense mechanism. Additionally, intravenous recombinant angiotensin converting enzyme 2 (ACE2) was recently reported as an effective therapy in severe COVID-19, by blocking viral entry, and thus reducing lung injury. Very few studies exist on the prognostic value of endothelium-related protective molecules in severe COVID-19 disease. To this end, serum levels of eNOS, inducible (i) NOS, adrenomedullin (ADM), soluble (s) ACE2 levels, and serum (s) ACE activity were measured on hospital admission in 89 COVID-19 patients, hospitalized either in a ward or ICU, of whom 68 had ARDS, while 21 did not. In our cohort, the COVID-19-ARDS patients had considerably lower eNOS levels compared to the COVID-19 non-ARDS patients. On the other hand, sACE2 was significantly higher in the ARDS patients. iNOS, ADM and sACE activity did not differ. Our results might support the notion of two distinct defense mechanisms in COVID-19-derived ARDS; eNOS-derived nitric oxide could be one of them, while the dramatic rise in sACE2 may also represent an endogenous mechanism involved in severe COVID-19 complications, such as ARDS. These results could provide insight to therapeutical applications in COVID-19.

## 1. Introduction

Several vasoconstrictors and vasodilators are produced by the vascular endothelium, such as endothelin-1, angiotensin-2, nitric oxide, and prostacyclin. These vasoactive substances play a role in the regulation of the vasomotor tone, the recruitment and function of inflammatory cells, and the regulation of thrombosis [1]. In acute respiratory distress syndrome (ARDS), the balance is shifted from pulmonary vasodilators towards vasoconstrictors, resulting in increased pulmonary vascular resistance and pulmonary hypertension [2,3].

A damaged endothelial system is an underlying condition of the many complications experienced by COVID-19 patients. Endothelial cells act as a barrier that must be breached before progression to severe disease. Endothelial nitric oxide synthase (eNOS) is an enzyme abundantly expressed in a variety of cell types, including endothelial cells, which constitutively produces nitric oxide (NO) [4]. The increased mortality risk associated with diseases that have underlying endothelial dysfunction, such as ARDS, suggests that eNOS-derived NO could be an important defense mechanism. Indeed, NO, which is the main intracellular antiviral defense, has been shown to inhibit, amongst others, the severe acute respiratory syndrome coronavirus 1 (SARS-CoV-1) [5]. Experimental studies in various lung injury models have demonstrated that increased levels of eNOS-derived NO are associated with beneficial effects [6,7,8,9,10,11,12,13,14], while the high bioavailability of NO from inducible NO synthase (iNOS) may aggravate lung injury [15,16]. Persistently high NO levels may result in the formation of cytotoxic reactive nitrogen intermediates [17].

Adrenomedullin (ADM) is a circulating peptide hormone with vasodilatory effects. ADM is essential for endothelial barrier development and stability. Data from experimental models have shown that treatment with ADM could attenuate lung injury, possibly by inhibiting endothelial hyperpermeability, inflammation, and apoptosis [18,19].

Additionally, data from clinical and experimental studies have suggested the implication of the renin-angiotensin system (RAS) in the pathogenesis of lung injury. Angiotensin-converting enzyme (ACE), the central component of RAS, converts angiotensin I to angiotensin II and breaks down bradykinin, while its analogue, ACE2, converts angiotensin II to angiotensin (1–7). Angiotensin II is a powerful vasoconstrictor, with pro-fibrotic and pro-inflammatory functions, while angiotensin (1–7) is a vasodilator, with potent anti-apoptotic and anti-proliferative functions [20]. Pulmonary capillary endothelium-bound ACE activity is used as a measure of pulmonary endothelial function, whose reduction is considered an early sign of lung injury [21]. Serum ACE activity, on the other hand, is often elevated in sarcoidosis, and several other inflammatory diseases of the lung [22]. From the beginning of the pandemic, it was shown that SARS-CoV2 enters the host cell by binding to ACE2. The shed isoform of ACE2, soluble (s) ACE2 retains catalytic activity, but its precise role in viral entry is still unclear. Intravenous recombinant ACE2 was recently reported as an effective therapy in severe COVID-19, by preventing viral entry in target cells [23].

Very few studies exist on the prognostic and diagnostic value of endothelium-related vasoactive molecules in COVID-19 severe disease, while to the best of our knowledge no study has investigated their role in COVID-19-induced ARDS. To this end, we measured serum levels of eNOS, inducible (i) NOS, adrenomedullin (ADM), sACE2, and serum (s) ACE activity on hospital admission (within 24 h).

## 2. Results

**Figure 1:**
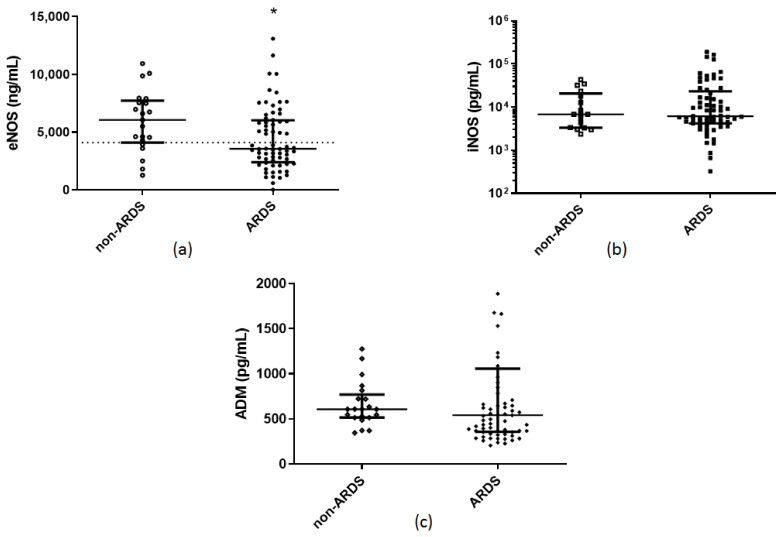
Hospital admission levels of soluble endothelium–related protective molecules in COVID-19 patients. (**a**) eNOS, (**b**) iNOS, and (**c**) ADM were measured in 21 COVID-19 non-ARDS patients and 68 patients with COVID-19-inflicted ARDS on hospital admission (within 24 h). Two–group comparisons were performed with the non-parametric Mann–Whitney test, * *p* < 0.05. Data are presented as scatter plots, indicating the median value and 25th to 75th centiles. Dashed line, median value of the whole cohort. ADM = adrenomedullin; ARDS = acute respiratory distress syndrome; eNOS = endothelial nitric oxide synthase; iNOS = inducible nitric oxide synthase.

**Figure 2:**
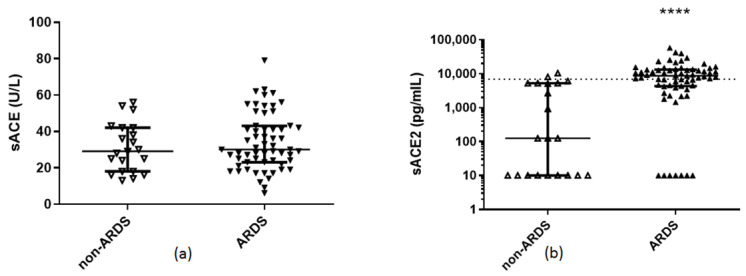
Hospital admission levels of serum ACE activity and soluble ACE2 in COVID-19 patients. (**a**) Serum ACE activity, and (**b**) soluble ACE2 were measured in 21 COVID-19 non-ARDS patients and 68 patients with COVID-19-inflicted ARDS on hospital admission (within 24 h). Two-group comparisons were performed with the non-parametric Mann–Whitney test, **** *p* < 0.0001. Data are presented as scatter plots, indicating the median value and 25th to 75th centiles. Dashed line, median value of the whole cohort. ARDS = acute respiratory distress syndrome; sACE = serum angiotensin converting enzyme; sACE2 = soluble angiotensin converting enzyme 2.

Demographics, clinical characteristics, and endothelial markers of the ARDS and non-ARDS patients are shown in Table 1. On hospital admission, ARDS patients had higher APACHE II and SOFA scores, C-reactive protein (CRP), and lactate dehydrogenase (LDH) levels. Most importantly, ARDS patients had decreased soluble eNOS levels and increased sACE2 levels, compared to non-ARDS patients (*p* < 0.05; Figure 1a and Figure 2b). In the ARDS patients, soluble eNOS and sACE2 exhibited a negative correlation (r_s_ = −0.295, *p* = 0.017). As expected, patients who required mechanical ventilation also had lower admission soluble eNOS levels (*p* < 0.01), and higher sACE2 levels (*p* < 0.01). It is worth mentioning that from the linear regression model applied, soluble eNOS and sACE2 levels were not dependent on age, sex, or comorbidities, and seemed to be statistically significantly dependent only on the presence of ARDS (*p* < 0.05). Finally, none of these circulating biomarkers correlated with a prolonged stay.

## 3. Discussion

To our knowledge this is the first study to report on the prognostic and diagnostic value of endothelium-related protective molecules in COVID-19-inflicted ARDS. Our results showed that in our cohort, the circulating levels of these biomarkers were altered in COVID-19-inflicted ARDS.

Circulating blood eNOS has been shown to contribute to the regulation of systemic blood pressure and the circulating NO pool [24]. In our COVID-19 cohort, patients with ARDS exhibited lower soluble eNOS levels, suggesting that higher eNOS activity, and the presumed increased NO synthesis, might protect patients from severe lung complications. The therapeutic use of inhaled (i) NO has been tested in COVID-19; iNO delayed respiratory deterioration in COVID-19-induced moderate to severe ARDS [25], whereas in another study it only minimally improved oxygenation in COVID-19-related ARDS [26]. On the contrary, it was not able to improve oxygenation in COVID-19 patients with refractory hypoxemia [27,28].

Studies on ADM have shown that at the mRNA and protein level, its levels were increased in patients with severe COVID-19 [29], and in COVID–19 induced endothelitis [30], respectively. In our COVID-19 cohort, ADM levels did not differentiate patients based on the presence of ARDS.

The SARS-CoV2 receptor for cell entry, ACE2, is deemed an ideal target for therapy. An engineered receptor has been shown to be catalytically active, and its close similarity with the native receptor has been suggested to limit the potential for viral escape [31]. Indeed, human recombinant sACE2 molecules have been shown to be effective in neutralizing COVID-19 infection by acting as decoy receptors [23]. These recombinant molecules can compete with ACE2 on endothelial cell surfaces for binding to SARS-CoV-2, leaving the membrane-bound ACE2 unoccupied and able to convert angiotensin II to angiotensin (1–7), thus moderating the overwhelming inflammatory response seen in COVID-19 [32]. A dramatic rise in sACE2 in one critically ill COVID-19 patient with ARDS, which preceded the recovery of the patient, prompted the authors to suggest that sACE2 represents an endogenous non-specific protective mechanism against SARS-CoV-2 infection [33]. In our COVID-19 cohort, sACE2 levels were much higher in severe disease. The therapeutic value of sACE2 has been suggested to exert a dual effect; a reduction in the levels of the vasoconstrictor angiotensin II, and blocking viral entry by masking the spike protein. However, in our cohort, as in other studies, severely ill patients, despite having high sACE2 levels, did not seem to gain a benefit in viral infection [34]. It has been proposed that a possible detrimental role of high sACE2 could be due to the binding of the virus to many sACE2 molecules; the virus-sACE2 complex can spread the virus to distant organs and create sACE2 and membrane ACE2 depletion. The resulting increased angiotensin II, part of the bigger pathology, might aggravate the disease [34]. It is thus of utmost importance to monitor both sACE2 and angiotensin II levels in COVID-19 patients to ensure the safe and efficient use of therapeutic sACE2. Of note, angiotensin II can downregulate eNOS protein expression via an angiotensin II type 1 receptor-linked pathway [35].

In our cohort of COVID-19 patients, serum ACE activity was similar in both groups. In a previous study, baseline serum ACE activity of severe and non-severe COVID-19 patients was decreased compared to normal controls; the lowest levels were noted in the severe group. The respective activities, however, increased in the recovery phase, and the authors concluded that serum ACE activity could be potentially used as a marker to reflect severity of COVID-19 at baseline [36]. In another study, no association was seen between serum ACE activity and COVID-19; furthermore, serum ACE activity on admission did not reflect disease severity [37].

The limitations of our study were the moderate sample size, and its single-center nature. We also measured ADM levels, which are typically underestimated due to rapid binding to its binding protein, fast metabolism, short half-life, and small concentrations; mid-regional pro-adrenomedullin is considered a better index. Additionally, we did not measure cytotoxic reactive nitrogen intermediates, due to the lack of suitable samples and/or laboratory techniques. Finally, measuring angiotensin II would provide us with further information on the vasoactive status of these patients.

## 4. Materials and Methods

This observational, single-center study included 89 consecutive COVID-19 patients, admitted either to the intensive care unit (ICU) of the “Evangelismos” Hospital (*n* = 67), or the specialized COVID-19 ward of our hospital (*n* = 22), from 22 March 2020 to 28 October 2020. SARS-CoV-2 infection was diagnosed by real-time reverse transcription PCR (RT-PCR) in nasopharyngeal swabs. The study was approved by the Research Ethics Committee of the “Evangelismos” Hospital (129/19–3–2020), and all procedures performed on patients were in accordance with the Helsinki Declaration. Informed written consent was obtained from all subjects or subjects’ next-of-kin.

Demographics, vital signs, comorbidities, symptoms, and laboratory measurements were recorded for each patient following enrollment. Acute physiology and chronic health evaluation (APACHE II) and sequential organ failure assessment (SOFA) score were calculated on ICU admission in the subset of critically ill COVID-19 patients, specifically in 60 ARDS and 7 non-ARDS patients.

The patients were categorized according to the presence of ARDS [38]. Four milliliters (4 mL) of venous blood were collected within the first 24 h post hospital admission, serum was prepared, portioned into 0.5 mL aliquots, and stored at −80 °C until used.

All biomarkers were assessed on hospital admission (within 24 h). Circulating levels of eNOS, iNOS, ADM, and sACE2 were measured by enzyme-linked immunosorbent assay (ELISA) (R&D Systems Inc., Minneapolis, MN, USA; Novus Biologicals, Bio–Techne Ltd., Abingdon, UK; Assay Genie, Dublin, Ireland). Serum ACE activity was determined by the ACE liquid photometric assay (Sentinel Diagnostics, Milan, Italy), on a Cobas c 702 module (Roche Diagnostics International AG, Rotkreuz, Switzerland). The detection limit of the assay is 2.4 U/L.

Data are presented as individual values, mean ± standard deviation (SD), or median with interquartile range (IQR). Comparison between groups was performed by the Student’s *t*-test, the non-parametric Mann–Whitney, or the chi-square test, as appropriate. Spearman’s correlation coefficient was used to assess correlations. A linear regression model was fitted to examine the relationship of age, sex, comorbidities, and the presence of ARDS on soluble eNOS and sACE2 levels. All tests were conducted using a Type I error, α = 0.05 and Type ΙI error β = 0.20 (80% power). The analyses were performed with the IBM SPSS statistical package, version 22.0 (IBM Software Group, New York, NY, USA), and GraphPad Prism, version 8.0 (GraphPad Software, San Diego, CA, USA). All the *p*–values were calculated after two-sided tests; *p*-values < 0.05 were considered significant.

## 5. Conclusions

In this moderate–sized COVID-19 cohort, soluble eNOS and sACE2 differentiated ARDS and non-ARDS patients. eNOS and sACE2 could, therefore, represent defense mechanisms in COVID-19-derived ARDS, providing insight into new therapeutical applications in COVID-19. Monitoring levels of vasoactive molecules is critical to ensure safe and efficient treatment with sACE2 and vaccines against components of the renin-angiotensin system.

## Figures and Tables

**Table 1:** Demographics, clinical characteristics, and endothelium-related protective molecules of hospitalized COVID-19 patients upon admission (within 24 h).

Characteristics	ARDS	Non-ARDS	P-value	Reference Values
Number of patients, N	68	21		
Age (years), (mean ± SD)	62 ± 13	61 ± 13	0.8	
Sex, N (%)			>0.9	
Male	53 (77.9)	17 (80.9)		
Female	15 (22.1)	4 (19.1)		
Comorbidities, N (%)	50 (73.5)	17 (80.9)	0.6	
ICU vs. ward, N (%)			<0.0001 *	
ICU	60 (88.2)	7 (33.3)		
Ward	8 (11.8)	14 (66.7)		
Sick days prior to admission, (mean ± SD)	7 ± 3	6 ± 4	0.2	
APACHE II, (mean ± SD) †	14 ± 5	8 ± 6	<0.0001 *	
SOFA, (median, IQR) †	6 (4–8)	3 (2–3)	<0.0001 *	
White blood cell count (per μL), (median, IQR) Neutrophils (%), (median, IQR) Lymphocytes (%), (median, IQR) Platelets (per μL), (median, IQR) CRP (mg/dL), (median, IQR) Fibrinogen (mg/dL), (mean ± SD) D-dimers (µg/mL), (median, IQR) LDH (U/L), (median, IQR) Ferritin (ng/mL), (median, IQR) Lactate (mmol/L), (mean ± SD)	8760 (5915–11,370) 83 (76–88) 12 (7–17) 225,000 (169,000–27,000) 11.5 (5.4–20.4) 630 ± 179 1.05 (0.46–2.29) 434 (339–574) 682 (265–2006) 1.5 ± 0.6	5630 (4355–12,405) 83 (63–87) 13 (9–29) 195,000 (155,250–269,500) 4.8 (1.6–11.1) 548 ± 151 0.74 (0.51–1.36) 257 (211–391) 376 (164–868) 1.4 ± 0.5	0.2 0.6 0.2 0.3 0.002 * 0.1 0.2 <0.0001 * 0.2 0.8	4–10.5 × 10^3^ 40–70 25–45 140–450 × 10^3^ <0.5 200–400 <0.5 <225 10–250 <2.0
Soluble endothelial–related molecules eNOS (ng/mL), (median, IQR) iNOS (pg/mL), (median, IQR) ADM (pg/mL), (median, IQR) sACE2 (pg/mL), (median, IQR) sACE (U/L), (median, IQR)	3570 (2405–6030) 6138 (4200–23,131) 543 (357–1057) 8700 (4313–13,325) 30 (23–43)	6070 (4025–7830) 6763 (3300–23,288) 608 (514–794) 125 (10–5225) 29 (18–42)	0.02 * 0.7 0.4 <0.0001 * 0.5	
Outcomes				
Mechanical ventilation, N (%)	50 (73.5)	4 (19.0)	<0.0001 *	
MV duration (days), (median, IQR)	8 (1–22)	0 (0–0)	<0.001 *	
LoS (days), (median, IQR)	18 (13–30)	8 (6–22)	0.002 *	
In–hospital mortality, N (%)	23 (33.8)	3 (14.3)	0.1	

* *p*–value < 0.05. Data are expressed as number of patients (N), percentages of total related variable (%) and mean ± SD for normally distributed variables and median (IQR) for skewed data. Patients were divided into two groups depending on the presence of ARDS on hospital admission. For differences between the two groups, either Student’s *t*-test for normally distributed data, the Mann–Whitney test for skewed data, or the chi-square test for nominal data was used. Characteristics were measured once (within 24 h of hospital admission). † APACHE II and SOFA score were calculated on ICU admission in 60 critically ill COVID-19 ARDS patients and 7 critically ill COVID-19 non-ARDS patients. Definition of abbreviations: ADM = adrenomedullin; APACHE II = acute physiology and chronic health evaluation II; ARDS = acute respiratory distress syndrome; CRP = C–reactive protein; eNOS = endothelial nitric oxide synthase; iNOS = inducible nitric oxide synthase; LDH = lactate dehydrogenase; LoS = length of stay; MV = mechanical ventilation; sACE = serum angiotensin converting enzyme; sACE2 = soluble angiotensin converting enzyme 2; SOFA = sequential organ failure assessment.

## Data Availability

Data are contained within the article.
